# Cell penetrating peptide: A potent delivery system in vaccine development

**DOI:** 10.3389/fphar.2022.1072685

**Published:** 2022-11-08

**Authors:** Behnam Hasannejad-Asl, Farkhondeh Pooresmaeil, Shahla Takamoli, Mehran Dabiri, Azam Bolhassani

**Affiliations:** ^**1**^ Department of Hepatitis and AIDS, Pasteur Institute of Iran, Tehran, Iran; ^2^ Department of Biotechnology, School of Advanced Technologies in Medicine, Shahid Beheshti, University of Medical Sciences, Tehran, Iran; ^3^ Department of Medical Biotechnology, School of Allied Medicine, Iran University of Medical Science, Tehran, Iran; ^4^ Department of Biology, Faculty of Science, University of Guilan, Rasht, Iran; ^5^ Department of Theriogenology, Faculty of Veterinary Medicine, University of Tehran, Tehran, Iran

**Keywords:** cell-penetrating peptides, protein transduction domains, CPP classification, physicochemical properties, vaccine, infectious and non-infectious diseases

## Abstract

One of the main obstacles to most medication administrations (such as the vaccine constructs) is the cellular membrane’s inadequate permeability, which reduces their efficiency. Cell-penetrating peptides (CPPs) or protein transduction domains (PTDs) are well-known as potent biological nanocarriers to overcome this natural barrier, and to deliver membrane-impermeable substances into cells. The physicochemical properties of CPPs, the attached cargo, concentration, and cell type substantially influence the internalization mechanism. Although the exact mechanism of cellular uptake and the following processing of CPPs are still uncertain; but however, they can facilitate intracellular transfer through both endocytic and non-endocytic pathways. Improved endosomal escape efficiency, selective cell targeting, and improved uptake, processing, and presentation of antigen by antigen-presenting cells (APCs) have been reported by CPPs. Different *in vitro* and *in vivo* investigations using CPP conjugates show their potential as therapeutic agents in various medical areas such as infectious and non-infectious disorders. Effective treatments for a variety of diseases may be provided by vaccines that can cooperatively stimulate T cell-mediated immunity (T helper cell activity or cytotoxic T cell function), and immunologic memory. Delivery of antigen epitopes to APCs, and generation of a potent immune response is essential for an efficacious vaccine that can be facilitated by CPPs. The current review describes the delivery of numerous vaccine components by various CPPs and their immunostimulatory properties.

## Introduction

Subunit and nucleic acids (NAs) vaccines are now the primary focus of vaccine development, rather than conventional vaccinations (Skwarczynski and Toth, 2016). The most significant benefit of subunit and NAs vaccines is that they have a better safety profile than conventional vaccines, which is the main issue in creating modern vaccines. These vaccines are created to contain only specific antigens, which are made up of antigenic peptides, polysaccharides, or proteins. This eliminates redundant parts and, as a result, lowers the risk of allergic or autoimmune reactions (Skwarczynski and Toth, 2014; Jaberolansar et al., 2016). An essential stage in the ability of vaccines to trigger immunological responses is the intracellular transport of antigens into antigen-presenting cells (APCs). The cell and organelle membranes are the main obstacle to transport of biologically active substances into target cells and various organelles. Diverse methods are employed for the trans-barrier delivery of various cargoes (Sánchez-Navarro, 2021). The exogenous materials must be transported to the target cells’ nucleus and cytoplasm to produce the protein products of the inserted gene and subunit vaccines. This procedure of transfection is performed through viral and non-viral vectors. The viral method is linked to increased technical demands but there are elevated risks of virus-related harm. Despite viral vectors, the non-viral vectors, such as nanocarriers and cell-penetrating peptides (CPPs), are significantly more affordable and simpler to manufacture in huge quantities. These vectors have restricted immunogenicity, which permits potential re-dosing. They are regarded safe since there is no possibility of recombination as detected in a competent virus that could potentially cause disease. CPPs, also known as protein transduction domains (PTDs), are the novel non-viral vectors that have attracted more considerate in the recent years (Lundberg and Langel, 2003).

To achieve efficient cell membrane translocation, CPPs were frequently fused with antigens. This improved antigen uptake, processing, and presentation by APCs increase both humoral and cellular immune responses. Without the aid of membrane proteins, CPPs can pass through the membrane semipermeable barrier and enter the cell interior non-invasively (Deshayes et al., 2005), but however, the delivery method of the CPPs and their cargo is not well known. Energy-independent (“direct penetration”) and endocytotic pathways have been proposed as the two prominent types of CPP absorption processes which can be influenced by the type and size of the CPP and cargo. Determining the precise absorption mechanism of a specific CPP/CPP-cargo is crucial for developing medication delivery systems based on CPP technologies. Over the past 10 years, CPPs have been widely used to enhance vaccine formulations and drug delivery systems since they are typically harmless and easy to produce economically (Yang et al., 2019).

Generally, CPPs were widely used for cargo delivery due to its high efficiency, good safety properties, and broad delivery of different cargoes. In addition, CPPs showed the advantages of low cytotoxicity and high penetration efficiency in various cell types. But however, CPPs possess some problems and limitations in clinical application such as: 1) Cellular uptake mechanism of CPPs is unclear; 2) Lack of cell and tissue specificity; 3) The penetration ability to different cells is variable; and 4) The stability needs to be improved (Zhang et al., 2021). Thus, despite main progress in design and application of CPPs, further studies are required to improve their delivery to different cells (*e.g.,* tumor cells), with reduced side-effects and enhanced therapeutic efficacy (Tripathi et al., 2018). Here, we will review the history and characteristics of CPPs, their applications as a non-viral delivery system for CPP-based vaccine development against infectious and non-infectious diseases, and at the end, we will study the different challenges of CPPs and their optimization methods.

### Overview of history and characteristics of CPPs

CPPs are small peptides and typically have 5–30 amino acids. To date, August 2022, the CPPsite 2.0 database, a user-friendly updated database that supplies different information about CPPs, reveals around 1700 unique, experimentally validated CPPs, together with their secondary and tertiary structures (https://webs.iiitd.edu.in/raghava/cppsite/). The human immunodeficiency virus (HIV) transactivating regulatory protein (TAT) was the first CPP that was raised in 1988 (Green and Loewenstein, 1988). A few years later, researchers discovered other naturally CPPs, such as the Herpes simplex virus (HSV)-1 protein-derived VP22 (Elliott and O'Hare, 1997), penetratin derived from the antennapedia, a *drosophila* homeoprotein (Derossi et al., 1994), transportan derived from a neuropeptide, *etc*. (Pooga et al., 1998). A variety of synthetic CPPs was later developed based on the structure of these naturally CPPs, including poly-arginine (Wender et al., 2000), poly-lysine (Heitz et al., 2009), model amphipathic peptide (MAP) (Oehlke et al., 1998), TP2 (Marks et al., 2011), MPG, *etc*. (Morris et al., 1997). Furthermore, *in silico* CPP predictions revealed the thousands of these peptides that are waiting to be verified and used. Based on physiochemical properties, CPPs fall under the following three broad classifications: 1) cationic, 2) amphipathic, and 3) hydrophobic (Lindgren et al., 2000). The positive charge of cationic CPPs has a strong affinity with the cytoplasmic membrane under normal physiological pH values. The negatively charged cell membrane glycoprotein combined with the cationic CPPs through electrostatic contact is subsequently internalized into the cell using a mechanism independent of the receptor. The first cationic CPP was Tat (Heitz et al., 2009). Positively charged peptides involve natural protamines and polylysine are employed as a vehicle for intracellular delivery of proteins (Haas et al., 2012) and NAs (Cotten et al., 1990). Research results from oligo-arginine cell penetration capability have revealed that increasing the number of arginines improves uptake capacity. Indeed, polylysine has a much weaker absorption profile in comparison with polyarginine (Tünnemann et al., 2008). Among the CPPs presently discovered, amphipathic CPPs, such as transportan (Zhang et al., 2019a) and MPG (Mehrlatifan et al., 2016), are the most usual, accounting for upper than 40%. They are peptides with both polar and non-polar properties, and the non-polar regions are rich in hydrophobic residues. These peptides target the membranes through interaction with the hydrophilic-hydrophobic nature of the lipid bilayer, thus displaying the properties of lipids’ hydrophilic and hydrophobic nature (Edwards et al., 2016). In contrast, there are just a few numbers of hydrophobic CPPs. These CPPs usually contain nonpolar/apolar residues or a functional group or hydrophobic motif that is essential for penetrating membranes. These peptides are generated by sequences of signal peptides containing residues of nonpolar peptides such as prenylates (Vickers, 2017), pepducins (Covic et al., 2002), and staples (Lau et al., 2015). Hydrophobic CPPs have a moderately low general charge, and the hydrophobic residues are important for membrane element interaction (Tian and Zhou, 2021). According to the findings, cumulative carrier hydrophobicity was a considerable determinant for improving the function of peptide in both internalization and activity of protein cargoes allowing for the finding of new and effective protein cargoes (Hango et al., 2021).

CPPs, due to their excellent efficacy and minimal toxicity, have become one of the most used techniques for accessing the intracellular space in recent years (Guidotti et al., 2017). Because of this capacity, they have been widely regarded as promising tools for intracellular delivery across the various bio-barriers including the blood-brain barrier (BBB), and intestinal, nasal and skin barriers (Shi et al., 2014; Zarei et al., 2020; Behzadipour et al., 2021). So, CPPs have been used in numerous biological and therapeutic contexts, including cancer and enzyme replacement treatment (Rapoport et al., 2011), vaccine development (Brooks et al., 2010), inflammation (Orange and May 2008; Lee et al., 2012), and diagnostic applications (Xie et al., 2020). The use of CPPs in the vaccine development against infectious and non-infectious diseases is one of the considerable favorable therapeutic processes.

### CPP classification

CPPs are classified based on their origin, conformation, and physical and chemical properties. Based on their origins, CPPs are divided into protein-derived CPPs (*e.g.,* Tat and penetratin), synthetic CPPs (*e.g.,* polyarginine), and chimeric CPPs (amphipathic peptide such as CADY). Based on their conformation, CPPs are divided into linear CPPs and cyclic CPPs. Based on differences in their physicochemical properties; CPPs are classified into cationic CPPs, amphipathic CPPs, and hydrophobic CPPs as shown in Supplementary Table S1. New CPP classification is based on the mechanisms of their entry into cells including direct penetration (*e.g.,* Barrel-Stave model, Carpet-like model, and the Inverted-micelle model), and one of several endocytic mechanisms (*e.g.,* macropinocytosis, caveolin-mediated endocytosis, clathrin-mediated endocytosis, and clathrin- and caveolin-independent endocytosis) (Xie et al., 2020; Yokoo et al., 2022; Zorko and Langel, 2022). Despite some common properties of CPPs (especially their cationic nature), their translocation mechanism is not similar for different families of CPPs. Generally, direct penetration is most probable at high CPP concentrations and for primary amphiphatic CPPs (*e.g.,* transportan analogues and MPG). The sequences of CPPs, their physicochemical properties, and the utilized experimental conditions are important in determination of the uptake mechanisms (Madani et al., 2011). Moreover, the uptake mechanism and efficiency were shown to be dependent on local peptide concentration, cell membrane lipid composition, characteristics of the cargo, and experimental methodology (Liu and Afshar, 2020). On the other hand, depending on the type of coupling to the cargo, CPPs can be classified into covalently or non-covalently bonded forms (Xie et al., 2020; Yokoo et al., 2022; Zorko and Langel, 2022).

### CPPs in vaccine delivery

Vaccination has progressively become common practice for the protection of all over the world from illnesses. Despite the achievements of traditional whole-organism vaccines, significant constraints prevent their widespread usage (Bull, 2015). For instance, the microorganisms in the live-attenuated vaccine might return to their virulent state and cause illness. In contrast, killed pathogens are unable to cause disease, but because of their poor immunogenicity, multiple booster doses are frequently necessary to elicit a strong enough immune response. Whole-pathogen-based vaccines frequently contain reactogenic components linked to unfavorable side effects with minor to fatal consequences. Additionally, because of the difficulty, or even incapacity, of growing some viruses (such as hepatitis B, hepatitis C, and human papillomavirus), standard vaccine designs are inapplicable for such diseases (Yang et al., 2019). For this reason, the focus of the current vaccine development has shifted away from conventional immunizations and toward nucleic acids (NAs) and subunit vaccines (Nevagi et al., 2018). NAs are highly charged anionic macromolecules that quickly removed from the fluid circulation. As a result, one of the most demanding issues in gene therapy is solving the problem of targeted delivery. Previously, viral vectors were utilized to transport NAs, but because to intrinsic immunogenicity, limited capacity to transfer macromolecules, and high cost of treatment, CPPs have progressively replaced them (Gaspar et al., 2020). Combining CPPs with NAs overcomes NAs’ weak permeability and stability while providing efficient intracellular delivery (Furukawa et al., 2020).

For the fusion of CPP with DNA vaccine, CPPs were generally complexed with DNA *via* non-covalent linkage, because the majority of CPPs have positively charged amino acids that can be easily attached to the negatively charged nucleic acids. Improvement in the immunogenicity of DNA vaccines following CPP delivery was demonstrated against viral infections. Moreover, CPPs were incorporated into other delivery systems loaded with DNA vaccines especially nanoparticles to enhance their intracellular delivery (Sadeghian et al., 2012). On the other hand, the potential of CPP/mRNA non-covalent complexation as delivery system was evaluated in cancer cells. The covalent-linking procedure is labor-intensive and time-consuming. Compared to the covalent strategy, the non-covalent method was easier to use, produce, and preserve the function of mRNA. CPPs complexed with antigen-encoding mRNA were successfully delivered to immune cells, and facilitated mRNA escape from the endosomes to the cytosol, inducing CTL responses (Kim et al., 2022). The lysine- and arginine-rich cationic peptides are able to form non-covalent complexes with nucleic acids but they require in most cases a covalent conjugation to proteins, marker molecules or drugs. Their positive charges favor the interaction with negatively charged components in the cell membrane and in the plasma. But, these CPPs are prone to be rapidly eliminated by reticulo-endothelial system (Reissmann, 2021).

Therapeutic peptides and protein domains, known as subunit vaccines, have enormous potential in vaccine development. They can exhibit good features, including selectivity due to broad interaction surfaces, stability under physiological circumstances, and tolerability. Generally, subunit and NAs vaccines are made up of pathogen components or their encoding genes. They are often non-immunogenic alone and thus an immunostimulating substance, like an adjuvant, must be included in their formulation. However, there are just a few approved adjuvants, and their immunostimulatory activity is frequently limited, while their toxicity might be significant (Nevagi et al., 2018). Nevertheless, this class of vaccine’s poor membrane permeability is a substantial impediment to their successful targeting of intracellular components. Many research companies have attempted to utilize CPPs in developing vaccine delivery systems during the last 10 years (JiangY, 2014). The goal is to transfer antigenic peptides into APCs.

Many vaccine delivery techniques have been developed to resolve these concerns. Most of them are intended to increase antigen stability *in vivo* and deliver them into the immune cells. CPPs are a particularly appealing component of antigen delivery systems (Yang et al., 2019) that often fused with antigens to facilitate effective cell membrane translocation and presentation by APCs. Also, they have been added to numerous NAs vaccine candidates to promote genetic material transport across plasma and nuclear membranes. Two pathways of exogenous and endogenous are involved for antigen presentation. Briefly, exogenous antigens are presented by the MHC-II molecules to CD4^+^ T cells leading to humoral immunity induction. In contrast, endogenous antigens are presented by the MHC-I molecules to cytotoxic CD8^+^ T cells (CTLs), which activate cellular immunity. In this line, CPP-antigen conjugates can be internalized by APCs through the non-endocytic pathway, and then they are degraded by proteasomes for presentation on the MHC-I molecules. Also, CPP-antigen conjugates can be internalized by APCs through endocytosis for presentation on the MHC-II molecules. Nucleic acids-based vaccines delivered by CPPs into the cells are translated to protein, and processed *via* the endogenous pathway (Sadeghian et al., 2012; Backlund et al., 2022). In general, CPPs promoted the vehicle of protein-based antigens and NAs cargo in both *in vitro* and *in vivo* experiments.

Current strategies for delivery of macromolecules such as nanoparticles, liposomes, viral-based vectors, microinjection, and electroporation may result in high toxicity, poor specificity, immunogenicity, and low delivery efficiency. As compared to these delivery strategies, CPPs can enter the cells in a non-invasive approach with high safety and potency (Xie et al., 2020). For instance, cell-penetrating peptides have proposed as promising tools for gene delivery. CPPs have emerged as strong tools for mRNA delivery in cancer therapy. The amphipathic CPP/mRNA complexes with a size less than 200 nm showed high cellular uptake and protein expression in tumor cells (Kim et al., 2022). Moreover, combinations of CPPs with lipid-based nanoparticles (LNPs) were followed in mRNA delivery (Yokoo et al., 2022). CPPs could improve endosomal escape efficiency, selective targeting of DCs, modulation of endosomal pathways for effective antigen presentation by DCs, and effective mRNA delivery to the lungs as well as prolong protein expression by intracellular stabilization of mRNA (Yokoo et al., 2022). Cationic peptides containing positively charged residues (lysine and arginine) could form complex with the negatively charged mRNA. They could stabilize the mRNA against degradation from serum RNases and protect mRNA vaccine from harsh storage conditions (Ramachandran et al., 2022). As a result, we hope that vaccine formulations based on CPPs, will enter clinical testing very soon.

### CPP-conjugated vaccines against infectious diseases

To improve the cellular uptake and effectiveness of preventative biomolecules against infectious illnesses, their conjugation with CPPs has been proposed and employed against bacterial, viral, and fungal diseases. In this section, we will study the usage of CPPs in novel vaccines against various infectious agents.

### CPP-conjugated antiviral vaccine

Despite the existence and effectiveness of a wide range of antiviral vaccines and medicines that can target viral components or different phases of the viral life cycle (Owji et al., 2020; Tompa et al., 2021), these compounds are only effective against a small subset of viruses. Additionally, significant adverse effects have restricted their widespread clinical utilization (Szunerits et al., 2015; Otręba et al., 2020). Therefore, the treatment and prevention of viral infections requires novel prophylactic and therapeutic approaches. NAs and peptides/proteins have been used extensively in recent years, as novel antiviral vaccines (Ptaszyńska et al., 2020).

Induction of strong cellular and humoral immune responses is necessary for adequate immunization against intracellular infections like viruses (Bungener et al., 2002; Casasola-LaMacchia et al., 2021). Numerous studies have shown that the CPP can enhance immune responses by increasing the transport and presentation of NA/protein/peptide-based antigens against viral infections. One example is the multiepitope peptide and protein vaccine development against HIV infection, the main reason of the acquired immune deficiency syndrome (AIDS), using MPG and HR9 CPPs. The results of immune responses demonstrated that these types of vaccine formulations significantly induced the secretion of antibodies, cytokines, and Granzyme B. Moreover, both CPPs had the same potency as a delivery system for stimulating immune responses (Davoodi et al., 2021). Hepatitis B virus (HBV), the principal cause of chronic hepatitis, cirrhosis, and hepatocellular cancer is another instance of vaccination’s prophylactic and therapeutic efficacy (Revill et al., 2020). For example, Chen et al. (2010) showed that immunization with TAT CPP fused to hepatitis B core antigen (HBcAg) greatly improved both humoral and cellular immune responses, produced strong specific CTL activity, and had therapeutic effects in HBV transgenic mice. On the other hand, CPPs such as Pep-1, Cady-2, p28, and hPP10 were used for delivery of E7 antigen in mice bearing human papillomavirus (HPV)-16-associated tumors. The results of vaccination showed that all the CPPs could facilitate E7 antigen uptake and among them, p28 CPP could significantly promote long-term protection against tumor challenge (Shahbazi and Bolhassani, 2018). Ji *et al.* designed intravaginal vaccines against HIV infection to overcome mucosal and epithelial barriers. Indeed, the antigenic HIVgag p24 gene added to the recombinant adenovirus (rAd) vector named as rAd/HIVgag-Tat-APS nanocomplexes induced HIVgag-specific CD8^+^ and CD4^+^ T cell responses, and higher levels of HIVgag-specific IgA and IgG in the vaginal cavity and serum in mice (Yang et al., 2019). The simultaneous use of M918 and MPG CPPs as protein and gene carriers improved HIV-1 Nef-specific B- and T-cell immune responses in mice, as well (Rostami et al., 2019). On the other hand, CPPs were studied to generate efficient vaccine against other viruses. Table 1 briefly shows CPP-based vaccines against viral infections.

**TABLE 1:** Examples of different CPP-conjugated vaccines against viral infections.

CPP	Cargo	Antigen/adjuvant	Virus	Vaccine strategy	Model	Effect	References
TAT, Pep-1, Cady-2	DNA, Protein	Nef/Gp96	HIV	Heterologous DNA prime/protein boost	Mice	• High cellular and humoral immune responses • Enhancement of T cell responses • High rates of IgG2a and IFN-γ directed toward Th1 responses, and also CTLs activity	Kadkhodayan et al. (2017)
TAT	Peptide	LCP-1	GAS	Homologous peptide prime/peptide boost	Mice	• High antibody titers • Increased opsonic activity	Yang et al. (2021)
PEI 600-TAT	DNA	E7	HPV	Homologous DNA prime/DNA boost	Mice	• Enhancement of cellular and humoral immune responses • Elevated Th1 response	Bolhassani et al. (2009)
MPG	DNA	E7	HPV	Homologous DNA prime/DNA boost	Mice	• Induction of a powerful Th1 cellular immune response with a prevailing IFN-γ profile	Saleh et al. (2015)
MPG	DNA	Core or coreE1E2/Montanide 720	HCV	Homologous DNA prime/DNA boost	Mice	• High levels of IgG1 and IgG2a isotypes • IFN-γ secretion in low concentrations •Development of Th1 immune responses	Mehrlatifan et al. (2016)
MPG, HR9, CyLoP-1, LDP-NLS	DNA, protein	Nef-Vpr- Gp160-P24 multiepitope construct	HIV	Heterologous DNA prime/protein boost	Mice	• Elevated secretion of Granzyme B, IFN-γ, IgG2a and IgG2b • Direction of immune responses toward Th1 and CTL activity	Davoodi et al. (2021)
	DNA, Protein	Nef-Rev- Gp160-P24 multiepitope construct	HIV	Heterologous DNA prime/protein boost	Mice	• High rates of Granzyme B and IFN-γ secretion, and low amounts of IL-10 secretion • High levels of IgG2a and IgG2b secretion	Shabani et al. (2022)
MPG, HR9, CyLoP-1, LDP-NLS	DNA, Protein	Nef-Vif-Gp160-P24 or Nef-Vpu-Gp160-P24 multiepitope constructs	HIV	Heterologous DNA prime/protein boost	Mice	• High levels of antigen-specific IgG2a and IgG2b responses • Elevated levels of Granzyme B and IFN-γ secretion • Direction of immune responses toward Th1 immune responses	Kardani et al. (2020)
MPG, M918	DNA, Protein	Nef/sHsp20	HIV	Heterologous DNA prime/protein boost	Mice	• High rates of Granzyme B, IFN-γ, IgG2a, and IgG2b secretion directed toward Th1 responses • Increased immune responses against HIV-1 Nef antigen	Rostami et al. (2019)
HR9, Cady-2	DNA, Protein	NS3/Hsp27	HCV	Heterologous DNA prime/protein boost	Mice	• Induction of a predominant IFN-γ, IgG2a, IgG2b profile with a high Th1 cellular immune response, and strong Granzyme B secretion	Alizadeh et al. (2019)
MPG, hPP10	DNA, Protein	E7/Hsp27 or Hsp20	HPV	Heterologous DNA prime/protein boost	Mice	• Induction of the E7-specific T cell responses	Bolhassani et al. (2018)
Pep-1	Protein	E7	HPV	Homologous protein prime/protein boost	Mice	• High Th1 cellular immune response with the predominant IFN-γ and IgG2a levels	Mardani et al. (2016)
LALF_32–51_	Protein	E7	HPV	Homologous protein prime/protein boost	Mice	• Ameliorate the presentation of E7-derived peptides to T cells	Granadillo et al. (2011)
Tat _47–57_	Protein	E7/CFA and IFA	HPV	Homologous protein prime/protein boost	Mice	• High titer of antibody • High frequencies of E7-specific CD8+T cells • Secretion of IFN-γ and CD107a expression • Long-term life span in animal model	Mousavi et al. (2021)

Abbreviations: CFA, complete freund’s adjuvant; CTL, cytotoxic T lymphocyte; GAS, group A *streptococcus*; Gp96, glycoprotein 96; HCV, hepatitis C virus; HIV, human immunodeficiency virus; HPV, human papillomavirus; Hsp27, heat shock protein 27; IFA, incomplete Freund’s adjuvant; IFN-γ, interferon-gamma; LALF_32–51_, the *Limulus polyphemus* protein; sHsp20, small heat shock protein 20; NS3, non-structural protein 3.

### CPP-conjugated antimicrobial vaccine

A big challenge in developing antimicrobial biomolecules against various microbial agents is multi-drug resistance (MDR). It has been demonstrated that DNA vaccination with transgene-expressing plasmids can trigger an immune response against multi-drug resistance. However, a delivery method and/or immunological adjuvant are required to administer these vaccinations. Yu *et al.* developed a recombinant DNA vaccine (Yu et al., 2016) encoding OprF gene (an antigenic surface protein) fused to HSV-1 VP22 CPP against *Pseudomonas aeruginosa* (*P. aeruginosa*) infection. The recombinant DNA vaccine harboring VP22 (pVAX1-OprF-VP22) considerably increased the levels of OprF-specific antibodies, and the survival rates in mice as compared to the recombinant DNA vaccine without VP22 (pVAX1-OprF) (Tompa et al., 2021). Furthermore, subunit vaccine development represents a profitable approach for preventing and treating various MDR microbial infections. Yang et al. (2021) utilized CPPs to produce a lipopeptide-based anti-group A *streptococcus* (GAS) vaccine. TAT (aa 47–57) or KALA CPPs were conjugated to multilamellar liposomes carrying LCP-1 antigen (*i.e.,* LCP-1/liposomes/TAT_47-57_ or LCP-1/liposomes/KALA), and their immunostimulatory potential was studied following intranasal administration in mice. The data showed that both constructs could elevate antibody titers, and offer high opsonic activity against clinically isolated GAS strains GC2 203 and D3840 (Yang et al., 2021).

*Mycobacterium tuberculosis* (MTB) infection remains a significant reason of morbidity and mortality worldwide (World Health Organization, 2013). The current vaccine, *mycobacterium bovis* Bacille Calmette-Guérin (BCG) has failed to prevent the adult pulmonary *tuberculosis* (TB) epidemic with a broad range of efficiency (Ritz et al., 2012). Thus, more influential vaccines are urgently required to prevent disease. In an attempt to generate an anti-tuberculosis subunit vaccine, Dong *et al.* generated a plasmid expressing the recombinant TAT-Ag85B fusion protein. Ag85B is the main protein secreted by all *Mycobacterium* species and is responsible for influencing defensive responses against MTB. Mice immunized with the recombinant TAT-Ag85B fusion protein produced higher levels of antigen-specific total IgG, IgG2a, and cytokines (TNFα and IFN-γ) than mice immunized with the recombinant Ag85B protein about 5 months after the last vaccination. Moreover, the MTB H37Rv (a virulent MTB strain) load was remarkably decreased in TAT-Ag85B-treated mice compared to that in Ag85B-vaccinated mice (Dong et al., 2015).

*Helicobacter pylori* (*H. pylori*) infection is intensely related to peptic ulcers, chronic gastritis, and gastric cancer. Antibiotic resistance in *H. pylori* is a progressively intense danger to global public health. Although, oral vaccination is believed to be a profitable strategy for defense against *H. pylori* infection, their poor effectiveness remains a considerable challenge because of their inadequate potency to penetrate mucus, and transit transepithelial absorption barriers (Lycke, 2012; Lopes et al., 2014). Zhang et al. (2018) generated a well-designed nanoparticle that is electrostatically self-assembled with CPP and antigen, and then coated with a “mucus-inert” polyethylene glycol (PEG) derivative. They indicated that polyarginine-containing nanoparticles loaded with the recombinant urease subunit B (rUreB) are more effective in triggering humoral immune responses against *H. pylori* infection than rUreB antigen sole upon oral delivery (Zhang et al., 2018).

Leishmaniases are disregarded diseases provoked by infection with *Leishmania* parasites, and there are presently no preventive vaccines against these parasites. A cell-mediated immune response against *Leishmania* is considered to defend against infection (Rodrigues et al., 2016). One of the most promising therapeutic processes is the use of CPPs in the dendritic cell (DC)-based vaccine (*i.e.,* CPP-antigen-based DC vaccination). CPP conjugation to antigens would improve DC uptake, antigen processing, and presentation on MHC class I and MHC class II molecules, conducting antigen-specific CD4^+^ and CD8^+^ T cell responses (Tacken et al., 2007; Palucka and Banchereau, 2012). For instance, TAT CPP fused to *Leishmania* homolog of receptors for activated C kinase (LACK) was used to induce immune responses against Leishmaniasis *via* DC vaccination. The TAT-LACK-pulsed DCs triggered the higher proliferation of CD8+T cells and IFN-γ releasing Th1 or T cytotoxic type 1 (Tc1) cells than LACK-pulsed DCs, showing that TAT increased the efficiency of vaccination (Kronenberg et al., 2010).

### CPP-conjugated vaccines against non-infectious diseases

The capacity of CPPs to translocate across membranes plays a vital role in the treatment of various non-infectious disorders such as ophthalmic illnesses (Liu et al., 2014; Tai et al., 2017; Pescina et al., 2018), central nervous system (CNS) disorders (Kim et al., 2015), inflammation (Derakhshankhah and Jafari, 2018; Kim et al., 2018), cancer, *etc*. Although CPPs have shown a positive potential in the context of drug delivery in a variety of non-infectious disorders, it seems that their use in the vaccine development has been limited to cancer. Due to growing advancement in this area, it is expected that CPPs are employed in the development of vaccines for other non-infectious disorders shortly.

Cancer is still one of the worst diseases, accounting for most fatalities globally. Thereby, finding the appropriate therapeutic strategy has high importance. In addition to using small molecule medications, macromolecules including proteins, monoclonal antibodies, NAs, and their combinations are now being used as anti-cancer vaccines. Despite many benefits, a significant difficulty still exists in the bio-distribution and translocation of these hydrophilic macromolecular medicines. Recent advancements in CPP have allowed the direct transfer of macromolecules into cells (Feni and Neundorf, 2017; Zhou et al., 2022). Pouniotis et al. (2006) and Pouniotis et al. (2016) demonstrated that penetratin in conjunction with cytotoxic T lymphocyte epitopes derived from ovalbumin or mucin-1 tumor-associated antigens could stimulate CD4^+^ and CD8^+^ T cells *in vitro*. Also, T cell-mediated cytokine release limited B16.OVA melanoma cell proliferation *in vivo*. On the other hand, pre-immunization with penetratin-OVA protected mice against a later tumor invasion.

Wu et al. (2019) developed a new kind of cancer DNA vaccine by combining a special CPP, cytosol-localizing internalization peptide 6 (CLIP6), with the model antigen ovalbumin (OVA) and CpG as an adjuvant. As compared to the naked OVA, the CLIP6-OVA conjugates exhibited a higher DC uptake, an enhanced antigen cross-presentation, and a stronger cytotoxic T lymphocyte (CTL)-driven immune response. They showed that the CLIP6-OVA/CpG formulation could elicit robust antigen-specific immune responses to slow the progression of challenged B16-OVA tumors in mice. Furthermore, such a CLIP6-OVA/CpG formulation is capable of acting as a therapeutic vaccination when coupled with PD-1 immune checkpoint blockade leading to considerable tumor regression for previously existing tumors. Until now, CPPs have been extensively used in many anti-cancer therapy approaches that make them a fantastic potential option for cancer treatment. CPPs could promote DC uptake of antigen peptides *in vitro*, increase vaccine immunogenicity in animal models, and improve the antitumor potency of cancer vaccines in mice. Early clinical trials of antigen-CPP vaccines are ongoing (clinicaltrials.gov: NCT04046445) (Backlund et al., 2022). Table 2 offers the examples of CPPs-conjugated vaccines in different cancers. Some CPPs were used with other nanocarriers for increasing cell specificity. For instance, pH-sensitive CPP-mediated endocytosis of nanocarriers was performed by replacement of the positively charged amino acids (lysines) of the CPP sequence by protonable ones (histidine) to convert it into a pH-sensitive CPP especially in tumor cells due to acidosis of tumor tissues (*e.g.,* high efficiency of TP10 CPP for DNA delivery after changing lysine residues to histidine residues). On the other hand, redox-responsive CPP-modified nanocarriers (nanocarriers with reduction-responsive units) have been often used for tumor-targeted delivery due to higher glutathione (GSH) concentration in tumors *versus* normal tissues (Sawant et al., 2013; Perche, 2019).

**TABLE 2:** Examples of different CPP-conjugated vaccines against cancers.

CPP	Cargo	Antigen/adjuvant	Cancer	Vaccine strategy	Model	Effect	References
CLIP6	Protein	OVA/CpG	B16-OVA melanoma	CLIP6-OVA protein along with CpG adjuvant	Mice	Increased uptake by dendritic cells—Enhanced antigen cross-presentation eliciting stronger cytotoxic T lymphocyte-mediated immune responses	Wu et al. (2019)
MPG^ΔNLS^	Protein	OVA/CpG	EG7-OVA tumor	MPG^ΔNLS^-OVA-loaded PLGA nanoparticles	Mice	The expansion of OVA-specific T-cells—Generation of OVA-specific IgG—Proliferation of OVA-specific memory T cells - Suppression of tumor growth and prolonged survival periods of the mice	Liu et al. (2019)
AntpMAPMUC1tet	DNA	CpG	MUC1^+ve^ melanoma	AntpMAPMUC1tet along with CpG	Mice	Enhanced antigen-specific interferon-gamma (IFN-γ) and IL-4 T cell responses—Induced Th1 response—Generation of long-term MUC1-specific antibody and T cell responses and delayed growth of MUC1^+ve^ tumors in mice	Brooks et al. (2018)
Polyarginine	Protein	FOXM1 N-terminal domain	Breast cancer	A recombinant FOXM1 N-terminal domain (1-138aa) fused with a nine arginine cell-penetrating peptide	Mice	Decreased the proliferation and migration abilities of cancer cells through binding to FOXM1 and FOXM1-interacting factor SMAD3 Prevention of tumorigenicity of cancer cells and inhibition of tumor growth in nude mouse xenograft models with no obvious signs of toxicity	Zhang et al. (2019b)
Arg_8_	Protein	OVA/Freund’s adjuvant	----	Cys-Trp-Trp-Arg_8_-Cys-Arg_8_-Cys-Arg_8_-Cys/OVA	Mice	Increased IgG titer and secretion of IFN-γ, IL-12, IL-4, and IL-10 cytokines Activation and maturation of dendritic cells	Wang et al. (2018)
Tat_49-57_	DNA	Survivin/IL-15	fatal colon carcinoma	Tat_49-57_/CTL epitope peptide surviving _85–93_/plasmid encoding murine IL-15	Mice	A robust memory CTL-mediated long-term response—Improving the survival rate	Yang et al. (2008)

Abbreviations: AntpMAPMUC1tet, mucin 1 (MUC1) variable number of tandem repeat (VNTR) containing multiple T cell epitopes and tetanus toxoid universal T helper epitope peptide (tetCD4); CLIP6, cytosol-localizing internalization peptide 6; CpG, unmethylated cytosine-guanine dinucleotides; MPG^ΔNLS^, a mutated version of MPG; OVA, ovalbumin; PLGA, poly (lactide-co-glycolide) acid; Arg_8,_ arginine octamer; IL-15, interleukin-15.

## Challenges of using CPPs and suggested solutions

Despite the high potential of CPPs in vaccine delivery, the FDA has not yet licensed any CPP-conjugated compounds, but numerous current clinical trials in various phases are studying them. These peptides have a variety of significant limitations that restrict their usage as mentioned below.

1) The primary problem with CPP clinical use is non-specificity. Practically every cell or tissue in the body can be exposed to CPP or CPP-cargo conjugate. Several delivery methods require CPPs directed to a specific area for reducing side effects and increasing preventive and therapeutic efficacy. In vaccine development, CPPs were known as promising tools for gene delivery. CPPs designed to target tumor tissues are used in developing a novel class of mRNA delivery tools in cancer therapy. Indeed, the presence of hydrophobic moieties, amino acid composition, and structure of CPP play a major role in complex formation with mRNA and the transfection efficiency of cancer cells (Kim et al., 2022). This strategy is important to develop tumor cells-based vaccines against a variety of cancers. On the other hand, the reports showed that intratumoral injection is more efficient than subcutaneous delivery in suppressing tumor growth using improvement of the tumor microenvironment (Che et al., 2021). Thus, the use of cell-specific CPPs can be effective for delivery of antigens intratumorally.

**FIGURE 1:**
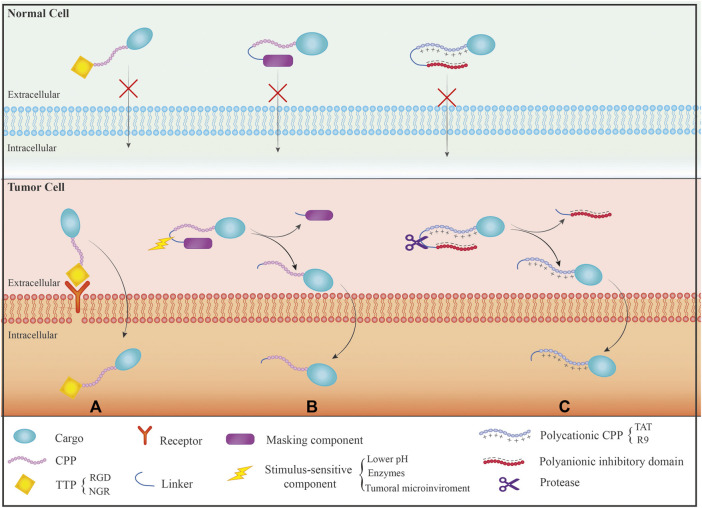
Different mechanisms of CPPs specificity on the normal and cancer cells: **(A)** The use of tumor targeting peptide (TTP) that has receptor only on the surface of tumor cells resulting in the effect of CPPs on specific target cells; **(B)** The use of activated CPP (ACPP) mechanism that causes activation of CPPs just in response to a motivating factor; **(C)** The use of polyanionic inhibitor and linker, which linker is only removed by the protease present in the target site, and then CPP interacts with the membrane to enter the cells.

Up to now, three general controlled delivery strategies have been proposed and developed to address the non-specificity problem of CPPs (Figure 1). These strategies include designing cell- and tissue-specific CPPs: A) Conjugating CPPs with targeting moieties and modulating CPP uptake by a stimulus-sensitive signal. These strategies aim to control CPP-cargo delivery only at the target site. Target-specific CPPs for cancer cells have been widely isolated using the phage display technique. Tumor targeting peptides (TTPs) are examples of this kind of peptides with high specificity and great affinity for a specific target (Kapoor et al., 2012). It is necessary to have a thorough understanding of the physicochemical features and internalization mechanisms of CPPs to rationally develop CPPs with high selectivity and efficiency; B) Targetable CPPs can be generated through conjugation with diverse cell-specific targeting ligands by covalent or noncovalent bonds. Folic acid (Meng et al., 2019), RGD peptides (Chu et al., 2017), transferrin (Yang et al., 2015), and antibody (Ye et al., 2015) are some examples of such targeting ligands; C) In the third and influential technique, various “smart” approaches have been devised to activate CPP function in a specific area of disease after systemic administration, utilizing various stimuli-responsive mechanisms (Huang et al., 2013). In this technique, activatable CPPs (ACPPs), stimulus-sensitive component, such as pH-sensitive, enzyme-sensitive, temperature-sensitive, electricity-sensitive, and light-sensitive materials, mask the CPP’s cell-penetrating function. When the CPP reaches a particular tissue environment, the ACPP receives a stimulator, the masking component is removed, and the CPP resumes its regular activity (Xie et al., 2020). In this respect, pH has been the most frequent factor in activating CPP at the target location, and pH-responsive peptides have frequently been used for therapeutic agent administration at the tumor site. Typically, tumor tissues have a lower pH than physiological pH (between 5.8 and 7.2) (Orange and May 2008). This method involves chemical modification or the use of pH-sensitive materials to reduce the capacity of CPPs to penetrate cells (Farkhani et al., 2014). The protected CPP recovers its capacity to penetrate cells once it reaches the acidic location (tumor site), leading to improved cellular entry into cancerous cells (Zhang et al., 2013). The development of such techniques is also possible using different triggers, including proteases, ultraviolet light, ultrasound, and temperature. As a sample, various ACPPs were created based on protease sensitivity. This method uses a polycationic CPP (such as R9 and TAT) whose cellular uptake is shielded by a polyanionic inhibitory domain that is covalently linked. When tissue-specific proteases (in the tumor tissues with elevated proteases) cleave the linker between the CPP and polyanionic inhibitory domain, the inhibitory domain will be removed, allowing the cleaved ACPP to enter cells. This method has mostly been applied to target the tumor tissues with a unique microenvironment containing hyperactive proteases (Zhu et al., 2012).

Generally, fusion or co-delivery of antigen with CPPs can increase antigen uptake, processing and presentation by antigen presenting cells (Yang et al., 2019). CPPs are an efficient system for cell-impermeable compounds to enter intracellular targets. Some CPPs indicated cell type specificity while some of them need modifications or form part of other delivery systems (*e.g.,* linkage of CPP to liposome or polymer). CPP-mediated strategies used to achieve cell selectivity include cell-penetrating-homing peptides, CPP-coupled cell-targeting peptide, targeting drug/cargo-coupled CPPs, directing CPPs using physical changes in the environment, and CPPs as a part of other delivery systems (Martín et al., 2010). These methods have been often used in drug delivery, but they can be applied in delivery of other cargoes such as vaccines.

CPPs can be classified into cell/tissue-specific and non-cell/tissue specific peptides (Zahid and Robbins, 2015; Taylor and Zahid, 2020). Cell/tissue-specific peptides (or transduction peptides) identified by screening of large peptide phage display libraries showed special potential in the diagnostic and therapeutic applications such as delivery of fluorescent or radioactive compounds for imaging, delivery of therapeutic peptides and proteins, and improvement of the DNA/RNA/siRNA/viral particles uptake (Zahid and Robbins, 2015; Taylor and Zahid, 2020). In addition, CPPs were used for immune modulation in several approaches including: 1) delivery of dominant-negative signaling molecules that can competitively inhibit the function of endogenous proteins in immune cells; 2) delivery of dominant negative molecules into cells; 3) intracellular delivery of negative regulators of key signaling pathways of the immune system; 4) chimeric peptide-based immune modulation by targeting transcription factors; 5) delivery of nucleotides into cells using CPPs for immune regulation (Lim et al., 2016). Although *ex vivo* DC manipulation has shown clinical success, but its potency has not been effective. It was shown that the use of CPP-conjugated antigens could overcome this disadvantage through enhancement of antigen delivery efficiency and long-time antigen presentation. For instance, DCs pulsed with CPP1-conjugated peptide antigen induced successful anti-tumor responses *in vivo* with higher efficiency than DCs pulsed with antigen alone. In fact, antigen delivery using CPP in the cytoplasm facilitated antigen presentation by MHC class I molecules leading to cytotoxic T lymphocyte activation likely through cross-presentation, and long-term immunological responses. These studies suggest that CPP can be used for DC vaccination against infectious diseases or various cancers, as well. Generally, the direct application of CPP-antigen for *in vivo* targeting has been successful in generating antigen-specific immune responses (Lim et al., 2016).

**FIGURE 2:**
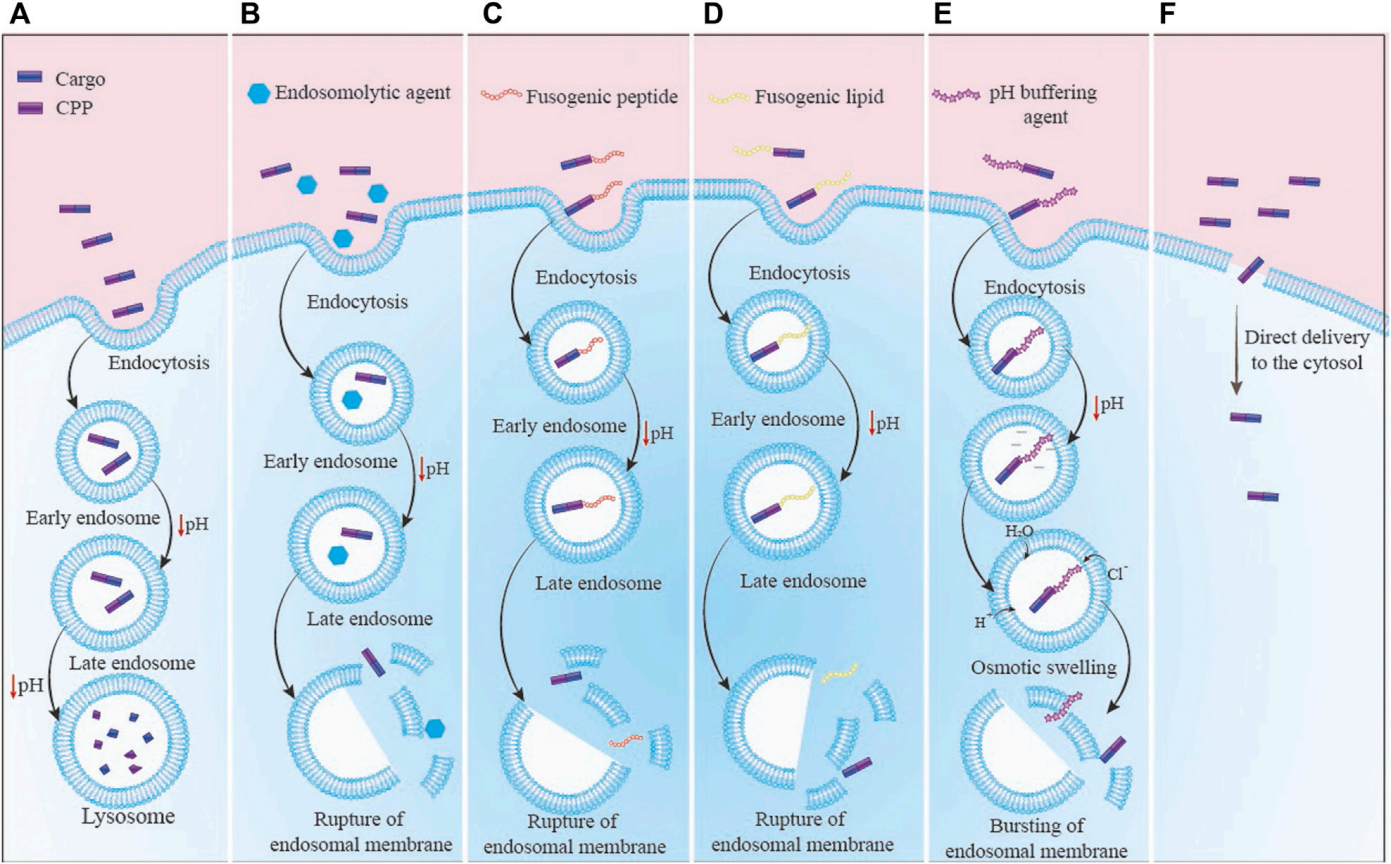
The CPP-cargo complex enters the cytosol through different routes: **(A)** Normal mode: the primary endosome has turned into a secondary endosome and finally into an endolysosome, which has led to the destruction of the complex; **(B)** Endosomolytic agents: There is an endosomolytic agent along with CPP that destructs the endosome membrane and results in the release of complex into cytosol space; **(C)** Fusogenic peptide: the fusogenic peptide is conjugated to CPP, which causes the degradation of the endosome membrane and the complex is released into the cytosol space; **(D)** Fusogenic lipids: Fusogenic lipid interacts with the inner surface of the endosome membrane and causes destruction of the membrane. Then, the complex is rescued into the cytosol space; **(E)** Proton sponge: In this mechanism, buffering agents linked to CPP-cargo complex or the residues available in CPP sequence like histidine enter water inside the endosomes. After endosome swelling and its destruction, the CPP-cargo complex is released into cytosol space; **(F)** Direct transfer of CPP: CPPs with a specific sequence lead to enter the CPP-cargo complex into the cytosol directly without the need for the endosome escape strategies.

**FIGURE 3:**
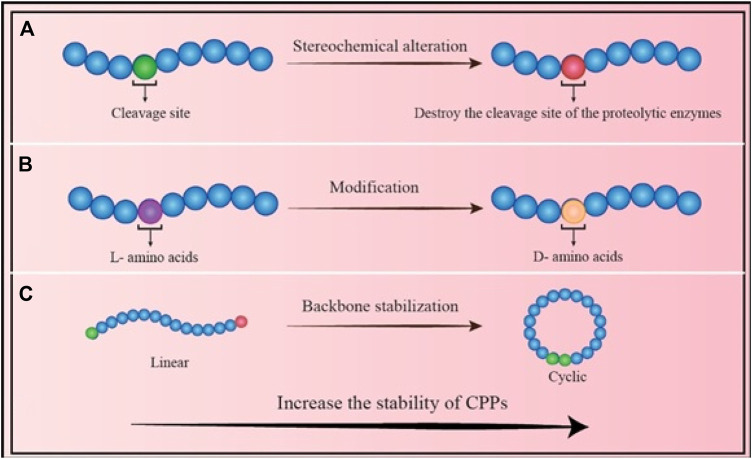
Different strategies to increase the stability of CPPs: **(A)** Stereochemical alteration method: Chemical modification of the residues eliminates the sensitivity of CPP; **(B)** Amino acid modification: Changing the residues to a different isomer can increase the stability of CPP; **(C)** Backbone stabilization: Converting the linear form to a circular CPP reduces the CPP’s sensitivity to enzymes, and increases its stability.

2) Another negative point of CPPs might be their ability to enter endosomes, which can result in the destruction of the CPP or its cargo by lysosomes (Erazo-Oliveras et al., 2012). For an effective CPP-based delivery, CPPs or CPP-cargo must successfully escape from the endosome and enter the cytosol before being degraded by lysosomes. There are various solutions to address this issue (Figure 2). A) One method is the combination of the CPPs with endosomolytic compounds such as sucrose, calcium, and chloroquine (Farkhani et al., 2014). In addition, some peptides known as fusogenic peptides including GALA peptide (Akita et al., 2010), melittin (Boeckle et al., 2006), and influenza virus hemagglutinin-2 (HA2) (Liou et al., 2012) have been used as noncovalent conjugation or as a covalent fusion with CPPs to enhance the endosomal escape of CPP complexes. These endosomolytic peptides alter their characteristics in reaction to the acidic endosome environment, which damages the endosome membrane (Martin and Rice, 2007); B) Another method to enhance the endosomal release of CPPs is the use of fusogenic lipids. By breaking the endosome membrane, fusogenic lipids like dioleoyl phosphatidylethanolamine (DOPE) have significantly accelerated cargo’s release from endosomes (El-Sayed et al., 2009); C) Other technique to boost the endosomal release of CPPs is the “proton sponge” effect. Histidine is a frequently employed compound that may be protonated to produce lysosome osmotic swelling (Beloor et al., 2015). In a research, TAT was coupled to polyhistidine (TAT-10H), and as a result, the histidine imidazole group, which has a pKa of 6.0, may function as a proton sponge in acidic endosomes (pH 5–6.5). Protonation of the histidine residues upon entrance into the endosome would result in osmotic swelling, endosomal membrane breakage, and release of CPP-cargo (Lo and Wang, 2008); D) Using the naturally existing or intelligently created CPPs capable of non-endosomal entrance is another strategy to prevent endosomal entry. The CPP or CPP-cargo can enter the cytoplasm without passing *via* an endosome in this method. The mechanism of CPP/CPP-cargo internalization was reported to be significantly dependent on many factors including type/concentration of CPP, type/size of cargo, type of cells, incubation time, charges, and experimental conditions (Shi et al., 2014). It was shown that endocytosis is the most typical uptake method at low CPP concentrations. At high concentrations, direct translocation is thought to be the primary main mechanisms for hydrophobic CPPs. Numerous cationic CPPs were discovered to have different uptake mechanisms based on the CPP concentration. Direct translocation is seen at concentrations over a certain threshold, but at lower concentrations, the endocytic pathway is the main route (Padari et al., 2010).
3) Another concern about CPP application is the potential toxicity and immunogenicity of CPPs. Most of the research examining CPPs *in vitro* has shown that they are non-immunogenic and have low toxicity. However, a comprehensive study, particularly *in vivo* analyses, is required due to the cationic character and origin of most CPPs, which might impact the integrity of the cell membrane and the possibility of triggered immune responses, respectively (Shi et al., 2014; Derakhshankhah and Jafari, 2018). In fact, the immunogenicity of CPPs can be affected by their various physicochemical properties including size, surface charge, amino acids sequence, hydrophilicity, morphology, and the type of conjugated cargo. However, the modified CPPs showed no specific immune responses (Zakeri-Milani et al., 2021). Generally, it is required to determine the non-toxic dose of CPPs. Although, fusion of CPPs or combination of them with vaccine/drug cargoes significantly reduces the toxicity of CPPs, but dose assessment will be valuable in drug and vaccine studies. This subject is important for immunogenicity of CPPs, as well.
4) Other drawback of CPPs is their proteolytic instability, which contributes to their limited plasma half-life. Due to their high instability and susceptibility to degradation, CPPs can be degraded by several protease enzymes that find in biological fluids such as blood, stomach or intestinal fluids, extracellular fluids, and intracellular fluids (Kristensen et al., 2016). Many strategies have been investigated to increase the stability of CPPs including A) altering their stereochemistry, B) modifying the specific amino acid residues, and C) changing their backbone (Figure 3). For instance, Nielsen et al.(2014) showed that switching a CPP from its L-form to its D-penetratin isomeric form significantly increased the half-life of the CPP in the intestinal fluid (Nielsen et al., 2014). Further analysis revealed improving the proteolytic resistance of octa-arginines (R8) by switching out L-arginine residues to D-arginine residues (Ma et al., 2012); In addition to stereochemical alterations, modifications can be made to individual amino acid residues, which can destroy the cleavage site of the proteolytic enzymes. TAT and MPG were modified to boost their proteolytic resistance while maintaining their ability to penetrate cells (Rennert et al., 2006); Another technique that might increase the durability of CPPs is backbone stabilization. Examples of such methods that increased the stability of CPPs against degrading enzymes include the incorporation of peptoid residues in the penetrating peptide sequence (Jing et al., 2012a; Jing et al., 2012b) and cyclization (Qian et al., 2016) of CPPs.

In addition to the factors mentioned above, it is crucial to thoroughly determine the biological distribution and pharmacokinetic characteristics of CPPs or CPP-cargo complexes in tissues and the circulatory system. In conclusion, the problems of CPPs must be resolved before they are used in clinical applications.

## Conclusion and outlook

CPPs, also known as trojan horse, are a hot topic delivery system in recent years. In addition to their ability to transfer across the cell membrane, they can deliver various prophylactic, therapeutic, and imaging agents as cargo. Because of their small size, CPPs were known as nanoparticles (NPs). The ability of NPs was evaluated as an appropriate delivery system in infectious and non-infectious diseases. One of the most prevalent non-infectious diseases is related to the CNS (central nervous system) disorders and tumors, which have more than one barrier for compound delivery including the BBB (blood-brain barrier) and cell membrane. Recently, various vectors such as gold NPs or Quantum Dots (QDs) help the BBB transfer of the biomolecules (Hasannejadasl et al., 2020; Hasannejad-Asl et al., 2022). But however, these vectors have limitations (Swain et al., 2016). CPPs as a novel NPs showed that they could transfer across the BBB from the transcytosis pathway without affecting the BBB and intracellular components of the barrier’s cells (Sharma et al., 2016), and then achieve the targeting cells in CNS. Derouazi *et al.* reported the development of a new class of recombinant protein cancer vaccines that deliver diverse CD4^+^ and CD8^+^ T-cell epitopes presented by MHC class I and class II alleles, respectively. In these vaccines, the recombinant protein was conjugated with Z12 CPP, which elevates effectual protein loading into the antigen-processing machinery of DC. Z12 prompted an integrated and multi-epitopic immune response with constant effector T cells. Treatment with Z12-formulated vaccines significantly extended survival in an orthotopic model of aggressive brain cancer. Analysis of the tumor areas exhibited antigen-specific T-cell accumulation with promising modulation of the balance of the immune infiltrate (Derouazi et al., 2015). In addition to non-infectious diseases, CPPs were used to deliver anti-infectious prophylactic and therapeutic vaccines against different microorganisms. For instance, immunization with CPP-conjugated HPV oncoproteins such as E7 and L2 had positive outcomes in the prevention of infection with HPV types 6, 11, 16, 18, and produced strong immunologic and anti-tumor responses in mouse model (Li et al., 2016; Shahbazi and Bolhassani, 2018).

CPPs were used as an appropriate delivery system; however, there are many limitations that hampered to CPP-based vaccines approved by FDA. To optimize the efficiency of CPPs and resolve their limitations, some methods were reported such as enhancement of the cell-type specificity, endosomal escape, and masking method. Jiao *et al.* designed the ch-Kn(s-s) R8-An micelles gene delivery system to dual-target the BBB and glioma using MMP-2-responsive peptides as the enzymatically degradable linkers. The glioma cells overexpressed low-density lipoprotein receptor-related protein-1 (LRP1), which specifically binds to the linker-conjugated angiopep-2. After the extracellular overexpressed MMP2 degraded the MMP2-cleavable linker and exposed the R8, the micelles could successfully target the glioma cells and enter the tumor’s center. This delivery method showed a high gene transfection efficiency in glioma cells (Jiao et al., 2019). Furthermore, scientists used different strategies like proton-sponge and disruptive peptides to endosomal-escape of CPP-cargo complex. The N-terminus of the HA2 fusogenic peptide forms α-helix structure that can be introduced into lipids. A conformational change in the low pH environment of endosomes causes the α-helix structure to fuse with the endosomal lipids, releasing CPP-HA2 complexes from the endosomes into the cytosol. Indeed, transducible TAT-HA fusogenic peptide increased escape of TAT-fusion proteins after lipid raft macropinocytosis (Wadia et al., 2004).

Overall, potent adjuvants are required in experimental vaccines based on proteins and peptides to trigger immune responses. An adequate replacement for current adjuvants involving MF59, ISCOMs, liposomes, *etc.,* will be the efficient translocation of proteins and peptides into APCs using CPPs (Foerg and Merkle, 2008). Additionally, unlike other targeting mechanisms for DNA delivery, CPPs can interface and bind to DNA vaccines directly without needing a poly-linker (Mwangi et al., 2005). (Cheng et al., 2001; Apostolopoulos et al., 2006; Rhee and Davis, 2006; Hou et al., 2013; Xu et al., 2016; Hajizadeh et al., 2020) Even though CPP-based vaccinations have many advantages over different types of vaccines including low toxicity, simple production, and non-immunogenicity in humans (Brooks et al., 2010), the *in vivo* stability, safety, improved cellular absorption, simplicity of synthesis, and cost of manufacture must also be considered in the ongoing efforts to offer these CPPs in clinics. It is recommended that sequences of CPPs resistant to protease action are used to improve the serum stability of these peptides while retaining the properties of CPPs.
