# Supplementary material for: Cell penetrating peptide: A potent delivery system in vaccine development
Source: Front Pharmacol. 2022 Nov 8;13:1072685. doi: 10.3389/fphar.2022.1072685 (PMC9679422; doi:10.3389/fphar.2022.1072685)
Supplement: Supplementary file 1 [file DataSheet1.pdf]

Supplementary Table 1: Examples of different CPPs based on their physiochemical properties

Type	CPP	Sequence	Origin	Cargo	Study model	Ref
Cationic	TAT (48-60)	GRKKRRQRRRPPQ	Protein-derived	siRNA	*In vivo*	(133)
	VP22	DAATATRGRSAASRPTERPRAPARSASRPRRVD	Protein-derived	RNA replicon vaccine against HPV16	*In vivo*	(134)
	Penetratin	RQIKIWFQNRRMKWKK-amide	Protein-derived	Peptide	*In vivo*	(135)
Amphipathic	Transportan	GWTLNSAGYLLGKINLKALAALAKKIL	Chimeric	siRNA	*In vivo*	(23)
	MPG	GALFLGFLGAAGSTMGAWSQPKKKRKV	Synthetic	Structural HCV genes	*In vivo*	(24)
	SV40 NLS	PKKRKV	Protein-derived	Plasmid DNA	*In vitro*	(136)
Hydrophobic	Melitin	GIGAVLKVLTTGLPALISWIKRKRQQ -amide	Protein-derived	siRNA	*In vitro*	(137)
	C105Y	CSIPPEVKFNPFVYLI	Protein-derived	Fluorescent label	*In vitro*	(138)

Abbreviations: HPV, human papillomavirus; HCV, hepatitis C; TAT, transactivating regulatory protein; NLS, nuclear localization signal
